# PTO-QuickStep: A Fast and Efficient Method for Cloning Random Mutagenesis Libraries

**DOI:** 10.3390/ijms20163908

**Published:** 2019-08-11

**Authors:** Pawel Jajesniak, Kang Lan Tee, Tuck Seng Wong

**Affiliations:** Department of Chemical & Biological Engineering and Advanced Biomanufacturing Centre, University of Sheffield, Sir Robert Hadfield Building, Mappin Street, Sheffield S1 3JD, UK

**Keywords:** cloning, QuickStep, PTO-QuickStep, random mutagenesis, directed evolution, protein engineering, synthetic biology

## Abstract

QuickStep is a cloning method that allows seamless point integration of a DNA sequence at any position within a target plasmid using only Q5 High-Fidelity DNA Polymerase and DpnI endonuclease. This efficient and cost-effective method consists of two steps: two parallel asymmetric PCRs, followed by a megaprimer-based whole-plasmid amplification. To further simplify the workflow, enhance the efficiency, and increase the uptake of QuickStep, we replaced the asymmetric PCRs with a conventional PCR that uses phosphorothioate (PTO) oligos to generate megaprimers with 3′ overhangs. The ease and speed of PTO-QuickStep were demonstrated through (1) right-first-time cloning of a 1.8 kb gene fragment into a pET vector and (2) creating a random mutagenesis library for directed evolution. Unlike most ligation-free random mutagenesis library creation methods (e.g., megaprimer PCR of whole plasmid [MEGAWHOP]), PTO-QuickStep does not require the gene of interest to be precloned into an expression vector to prepare a random mutagenesis library. Therefore, PTO-QuickStep is a simple, reliable, and robust technique, adding to the ever-expanding molecular toolbox of synthetic biology and expediting protein engineering via directed evolution.

## 1. Introduction

Molecular cloning has spurred progress throughout the life sciences [1,2]. In the late 1960s, several laboratories independently isolated host and bacteriophage-induced DNA ligases from *Escherichia coli* [3]. The first type II restriction enzyme from *Haemophilus influenza* (HindII) was isolated and characterized in 1970 [4]. These landmark experiments paved the way for gene cloning using restriction digestion and ligation, a popular method that is still widely and routinely used. As we move into the era of synthetic biology, the field is in need of low-cost, time-efficient, and more robust methods for high-quality DNA assembly [5]. QuickStep-Cloning was developed in response to this demand [6,7].

**Figure 1:**
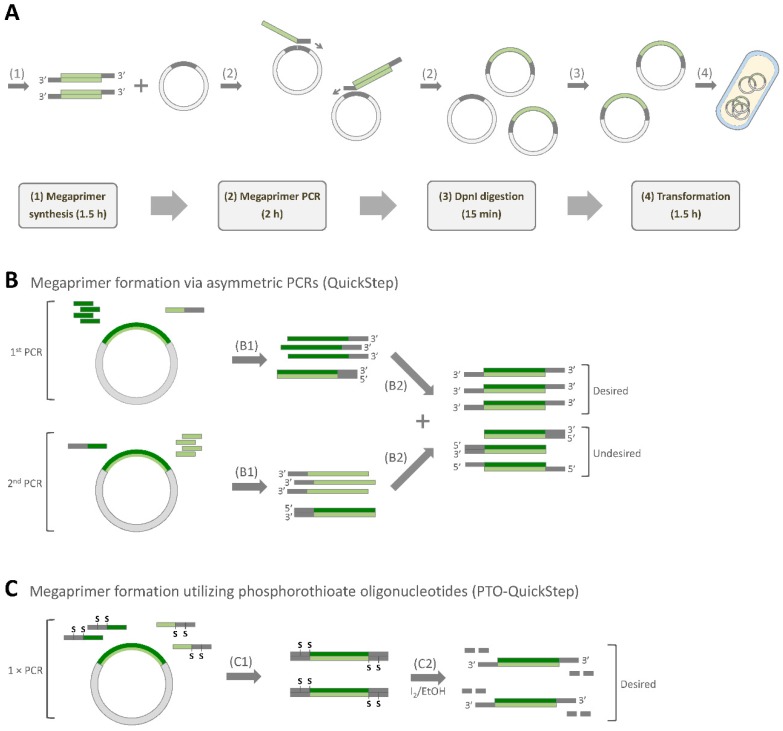
Overview of QuickStep and PTO-QuickStep. (**A**) Major steps of the method and estimated durations when cloning a 1 kb insert into a 7 kb recipient plasmid (adapted from [6,7]). (**B**) Molecular mechanism of megaprimer synthesis via two parallel asymmetric PCRs, featured in the original QuickStep method (B1: two asymmetric PCRs, B2: PCR purification and mixing of the products of the two asymmetric PCRs). (**C**) Molecular mechanism of megaprimer synthesis utilizing phosphorothioate (PTO) oligonucleotides, featured in PTO-QuickStep (C1: a standard PCR with PTO oligos, C2: iodine cleavage of PTO bonds via 5 min incubation in alkaline iodine solution. The short strands of DNA produced during the cleavage reaction are removed in a subsequent PCR purification).

QuickStep is a cost-effective method for high-efficiency gene cloning that uses only Q5 High-Fidelity DNA Polymerase and DpnI endonuclease. These two enzymes are commonly found in most research laboratories. QuickStep is a two-stage protocol, starting with the generation of megaprimers with 3′ overhangs using two parallel asymmetric PCRs, followed by an exponential whole-plasmid amplification (Figure 1A). The key advantages offered by QuickStep include an exponential amplification (higher efficiency), point integration of DNA fragment at any desired position (higher flexibility), compatibility with low-efficiency bacterial transformation methods (e.g., CaCl_2_ method), and time efficiency.

The megaprimer synthesis stage in QuickStep involves two parallel asymmetric PCRs (Figure 1B), which necessitate substantial preparation time. On top of that, megaprimers with 3′ overhangs are created by preparing an equimolar mixture of the two asymmetric PCR products. Ideally, each asymmetric PCR would produce only single-stranded DNA (ssDNA) as product, which forms a building block for the double-stranded megaprimer. In reality, the complementary strands with 5′ extension are also produced in each asymmetric PCR, albeit in a smaller amount. When the products of both asymmetric PCRs are mixed, these complementary strands with 5′ extension can either anneal (1) to 5′ extended strands, resulting in the formation of megaprimers with 5′ overhangs or (2) to 3′ extended strands, giving rise to blunt-ended megaprimers, as illustrated in Figure 1B. These two types of undesired double-stranded megaprimers are “nonproductive” as they cannot be extended in the subsequent whole-plasmid amplification stage. To eliminate these two problems (the need for two asymmetric PCRs and the presence of the undesired megaprimers) and further simplify the method, we proposed phosphorothioate (PTO)-QuickStep. In this streamlined protocol, we replaced the two parallel asymmetric PCRs with a single conventional PCR using PTO oligonucleotides (Figure 1C). 3′ overhangs were subsequently exposed by a short megaprimer incubation in alkaline iodine solution.

The PTO bond substitutes a sulfur atom for a nonbridging oxygen in the phosphate backbone of an oligonucleotide. PTO modification renders the internucleotide linkage more resistant to nuclease degradation [8], which explains why this modification is commonly employed in therapeutic oligonucleotides [9]. PTO bond also exhibits chemical stability similar to a phosphodiester linkage [9]. Another interesting feature of PTO bond is that it is susceptible to iodine cleavage in alkaline solution. Selective strand cleavage is achieved through alkylation to form the hydrolytically labile PTO trimester [10]. This specific feature has been exploited in the development of several molecular biology tools, such as sequence saturation mutagenesis (random mutagenesis) [11,12,13], OmniChange (focused mutagenesis) [14], and PLICing (gene cloning) [15].

In this article, we present the optimized PTO-QuickStep. We demonstrated the applicability of PTO-QuickStep by applying it to cloning of the gene encoding the reductase domain of cytochrome P450 BM-3 (*cpr*, ~1.8 kb). We further extended the method to create a random mutagenesis library of a red fluorescent protein (mRFP1). This was the first attempt of coupling PTO-QuickStep and random mutagenesis.

## 2. Results and Discussion

### 2.1. PTO-QuickStep and its Optimization

PTO-QuickStep was designed to streamline QuickStep-Cloning, which was reported previously by our group [6,7]. The objective was twofold: (1) to reduce the preparation time by replacing two parallel asymmetric PCRs with a single conventional PCR and (2) to remove unwanted by-products during megaprimer preparation (Figure 1B). Compared to QuickStep, which requires four primers, PTO-QuickStep utilizes only two oligonucleotides, each containing two PTO modifications (Figure 1C and Table 1). PTO bonds were selectively cleaved by adding alkaline iodine solution to expose the 3′ overhangs. We strategically incorporated two PTO modifications to build in a “fail-safe” mechanism in our design (Figure 1C). Even if only one PTO bond was cleaved (i.e., incomplete iodine cleavage), the subsequent exponential megaprimer-based whole-plasmid amplification would still work. On top of that, shorter DNA fragments (half the length) were generated from iodine cleavage when two PTO modifications were used, which facilitated the subsequent removal of cleaved DNA fragments via a silica-based PCR purification.

PTO-QuickStep was optimized using an *rfp* cloning experiment, where *rfp* gene from pBbA8k-RFP (Kan^r^; donor plasmid) was cloned into pET24a-HLTev-p53 (Kan^r^; recipient plasmid). This *rfp* gene encodes the monomeric mRFP1, originally derived from the *Discosoma* sp. red fluorescent protein (DsRed) [16]. For the first stage of PTO-QuickStep (megaprimer preparation), two parameters were investigated: (1) iodine cleavage time and (2) the effect of a PCR mix on the efficiency of iodine cleavage. For the second stage of PTO-QuickStep (megaprimer PCR), three parameters were studied: (1) megaprimer concentration, (2) number of PCR cycles, and (3) recipient plasmid concentration.

**Figure 2:**
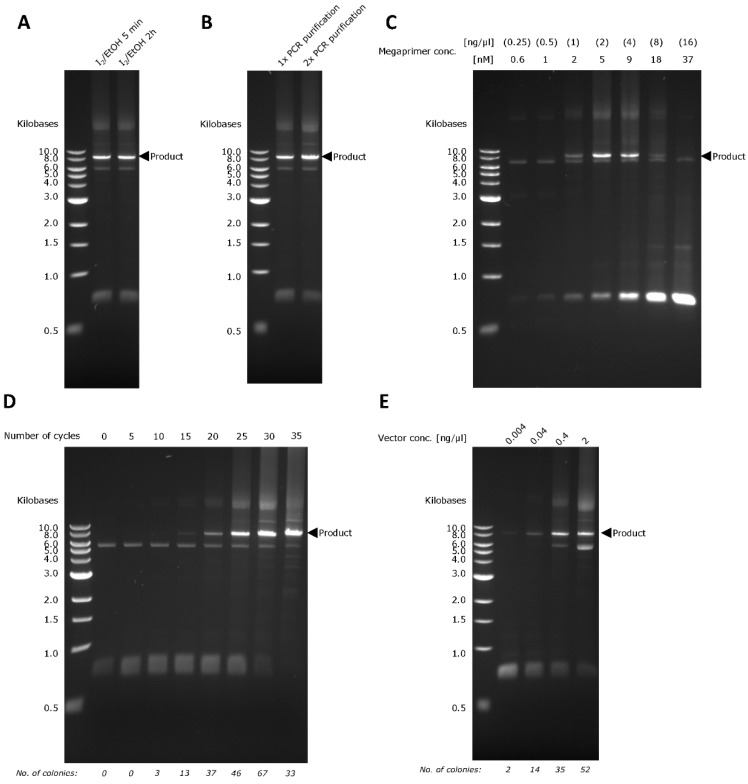
Optimization of PTO-QuickStep. The DNA gel shows the yield of megaprimer PCR (**A**) for different iodine cleavage times, (**B**) with and without an additional PCR purification step prior to iodine cleavage, (**C**) for different megaprimer concentrations, (**D**) for different number of PCR cycles, and (**E**) for different recipient plasmid concentrations.

After the PCR with PTO oligonucleotides, 6.25 µL of 0.5 M Tris-HCl buffer (pH 9) and 6.25 µL of 100 mM iodine in absolute ethanol were added directly to the PCR product (50 µL). After mixing, the mixture was incubated for 5 min at 70 °C and then snap-cooled on ice to prevent the cleaved DNA fragments from reannealing to the exposed 3′ overhangs. A quick silica column-based PCR purification was conducted before proceeding to the megaprimer PCR. This PCR purification step is necessary for two purposes: (1) to remove the cleaved DNA fragments, which are ssDNA molecules of 11–16 bases assuming a complete iodine cleavage of both PTO bonds (with a QIAquick PCR purification, only DNAs of 100 bp to 10 kb are purified) and (2) to remove ethanol that interferes with any downstream enzymatic reaction. As shown in Figure 2A, there was no discernible difference when the incubation time in iodine solution was extended from 5 min to 2 h. We also investigated whether there was a need to add an additional PCR purification step just before the iodine cleavage. It is clear from Figure 2B that an extra PCR purification step barely altered the final PCR yield. This indicated that the PCR mix did not interfere with the iodine cleavage, which was also observed in other studies [11,12,13].

Learning from our previous experience [6,7], the efficiency of exponential megaprimer-based whole-plasmid amplification is significantly influenced by megaprimer concentration, the number of PCR cycles, and recipient plasmid concentration. By varying megaprimer concentration between 0.25 ng/µL to 16 ng/µL, it was determined that the optimal PCR yield occurred at 2 ng/µL of megaprimer, which is equivalent to a concentration of 5 nM (Figure 2C). Generally, we observed higher PCR product yield with increasing number of PCR cycles (Figure 2D and Table 2). However, as we increased the number of PCR cycles, we also noticed more side-products (indicated by the smears above and below the product band; Figure 2D). For 35 cycles, the higher PCR product yield did not translate into higher cloning efficiency (Figure 2D). This was most likely caused by a high amount of side-products that interfered with the subsequent chemical transformation. The same trend was observed when we varied the recipient plasmid concentration. Higher PCR product yield was obtained when we increased vector concentration, which was also accompanied with a higher side-product formation (Figure 2E). Therefore, 2 ng/µL (5 nM) of megaprimer, 30 cycles, and 0.4 ng/µL of vector were chosen to be the most optimal conditions for a megaprimer PCR based on two considerations, namely, the amount of PCR side-products and the number of transformants obtained.

### 2.2. PTO-QuickStep is Superior to QuickStep-Cloning and RF Cloning

**Figure 3:**
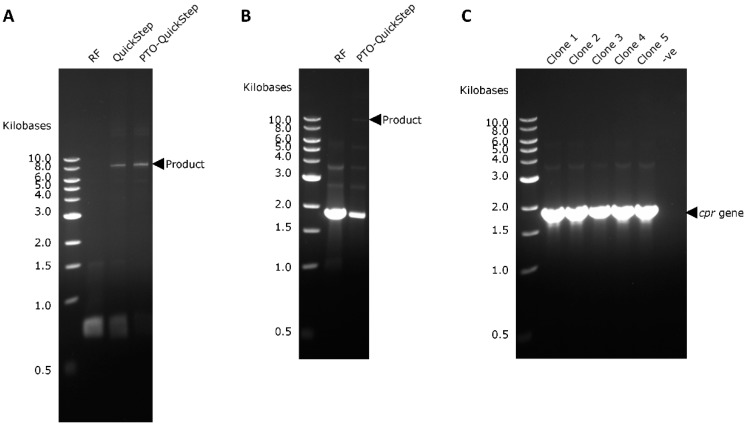
(**A**) Megaprimer PCR yield of RF cloning, QuickStep and PTO-QuickStep in the *rfp* cloning experiment. (**B**) Yield of RF cloning and PTO-QuickStep in the *cpr* cloning experiment. (**C**) Colony PCR confirming the presence of *cpr* gene in all five randomly selected clones from the cloning experiment using PTO-QuickStep.

The optimized PTO-QuickStep was then compared to QuickStep-Cloning [6,7] and restriction-free (RF) cloning [17] by conducting the same *rfp* gene cloning using identical primer design and recipient plasmid to allow an objective and fair assessment. All PCR products were transformed directly into *E. coli* C41 (DE3) strain for a T7-driven mRFP1 protein expression, using the standard CaCl_2_ method. As evidenced in Table 2, PTO-QuickStep was clearly superior in terms of efficiency and accuracy. Compared to QuickStep, PTO-QuickStep showed a 2- to 4-fold improvement in cloning efficiency, depending on the number of PCR cycles used. At the same time, the percentage of mRFP1-positive clones remained exceptionally high (84% for PTO-QuickStep and 100% for QuickStep). The low percentage of mRFP1-negative clones (potentially caused by deleterious mutations such as frameshift or point mutation) means that both PTO-QuickStep and QuickStep are characterized by a high degree of fidelity. On the other hand, RF cloning failed to produce a single colony. This was consistent with the observation that the megaprimer PCR of RF cloning did not yield a distinct PCR product band (Figure 3A). The unsuccessful RF cloning could be attributed to two factors: (1) RF cloning is not designed for point integration of a gene into a recipient plasmid, and (2) RF cloning requires a transformation method of much higher efficiency (e.g., electroporation or ultracompetent cells).

It is important to note that all methods tested in this study (PTO-QuickStep, QuickStep, and RF) generated nicked circular plasmids. After transformation, *E. coli* cells repaired the nicks in the plasmids. We also did a parallel transformation using intact circular plasmid (in this case, the recipient plasmid pET24a-HLTev-p53) as a reference. We achieved a transformation efficiency of 2 × 10^4^ cfu/µg (Table 2), which is equivalent to 20 colonies per 1 ng of intact plasmid. This benchmark was necessary for two reasons: (1) the number of transformants obtained in a gene cloning depends on the strain and the transformation method used, and (2) it is impossible to quantify the exact concentration of the desired product from a megaprimer PCR due to the presence of unavoidable side-products. The transformation efficiency of a nicked plasmid is widely known to be lower than that of an intact plasmid. This explains why the numbers of transformants from all cloning methods were lower than that of the reference plasmid.

### 2.3. Right-First-Time Cloning of P450 BM-3 Reductase Gene (cpr)

*rfp* (678 bp) is a relatively short gene. To prove that the optimized protocol is versatile and applicable to cloning other longer genes without the need for further optimization, we applied PTO-QuickStep to cloning P450 BM-3 reductase gene (*cpr*; 1782 bp) from pCWori-BM3 (Amp^r^; donor plasmid) into pETM11-BMP-WT (Kan^r^; recipient plasmid). This cloning was considered technically more challenging as the target gene (~1.8 kb) and the recipient plasmid (~7.4 kb) would give a combined product of ~9.2 kb. Typically, the number of transformants decreases with the increase in plasmid size. To serve as a comparison, we performed RF cloning concurrently. To ensure that any observed differences originate from the intrinsic molecular mechanisms underlying the two cloning methods, the number of cycles for megaprimer PCR was set to 25, which is the recommended number for RF cloning. Owing to the large product size and an insert that was nearly 3 times longer, the PCR product yield was much lower (Figure 3B) compared to the *rfp* cloning experiment (Figure 3A). Despite the low DNA amount, judging from the DNA gel, we obtained a significant number of transformants (Table 2). Five of the 49 colonies were randomly picked, and the presence of *cpr* gene was confirmed for all five clones (100% accuracy; Figure 3C). The clones were sequence verified and gave the expected P450 BM-3 activity (data not shown), proving the high fidelity of PTO-QuickStep. Once again, RF cloning did not produce a colony, further confirming our initial hypotheses that RF cloning is not designed for point integration of a gene and/or it requires a more efficient transformation method.

### 2.4. Applying PTO-QuickStep for Directed Protein Evolution

To maximize the potential of PTO-QuickStep, we applied the protocol to prepare a random mutagenesis library for directed evolution. *rfp* gene was once again used as a model system. However, this time, random mutations were introduced into *rfp* gene by amplifying the gene using PTO oligonucleotides and Taq DNA polymerase in the presence of imbalanced deoxynucleoside triphosphate (dNTP) concentrations and MnCl_2_. In other words, a standard error-prone PCR (epPCR) condition was used. After the epPCR and iodine cleavage, we proceeded to megaprimer PCR as per usual. Again, for a more objective comparison, 25 cycles of megaprimer PCR were used. Concurrently, we prepared another random mutagenesis library following the megaprimer PCR of whole plasmid (MEGAWHOP) protocol [18]. It should be noted that, in order to perform MEGAWHOP, the target gene (in this case, *rfp* gene) must first be precloned into the expression vector. This prerequisite would mean an extra step when applying MEGAWHOP compared to PTO-QuickStep. However, the advantage is a higher number of transformants due to better annealing of the megaprimers (the complementary region is essentially the entire target gene). When we transformed the products of both PTO-QuickStep and MEGAWHOP into *E. coli* C41 (DE3) using electroporation, we obtained comparable library sizes (Table 2), despite the fact that a suboptimal number of cycles (25 instead of 30) for megaprimer PCR was used for PTO-QuickStep to allow for an objective comparison between the two methods. Contrary to gene cloning experiments described above, electroporation was applied here to demonstrate that the number of transformants obtained (and consequently the library size in the case of a directed evolution experiment) can easily be upscaled through a more efficient transformation method. It is worth noting that a transformation efficiency of 2 × 10^6^ cfu/µg DNA was obtained using electrocompetent cells prepared in-house. This efficiency is far lower than that of commercially available ultracompetent cells (>1 × 10^9^ and >1 × 10^10^ cfu/µg pUC19 DNA for chemically competent and electrocompetent cells, respectively). As such, the library size can be further increased by more than three orders of magnitude by combining the product of PTO-QuickStep with commercial ultracompetent cells. Nonetheless, our data showed that both chemical transformation and electroporation are compatible with PTO-QuickStep. The choice is dependent on the number of transformants required and the application.

### 2.5. mRFP1 Variants Isolated from PTO-QuickStep Library

**Figure 4:**
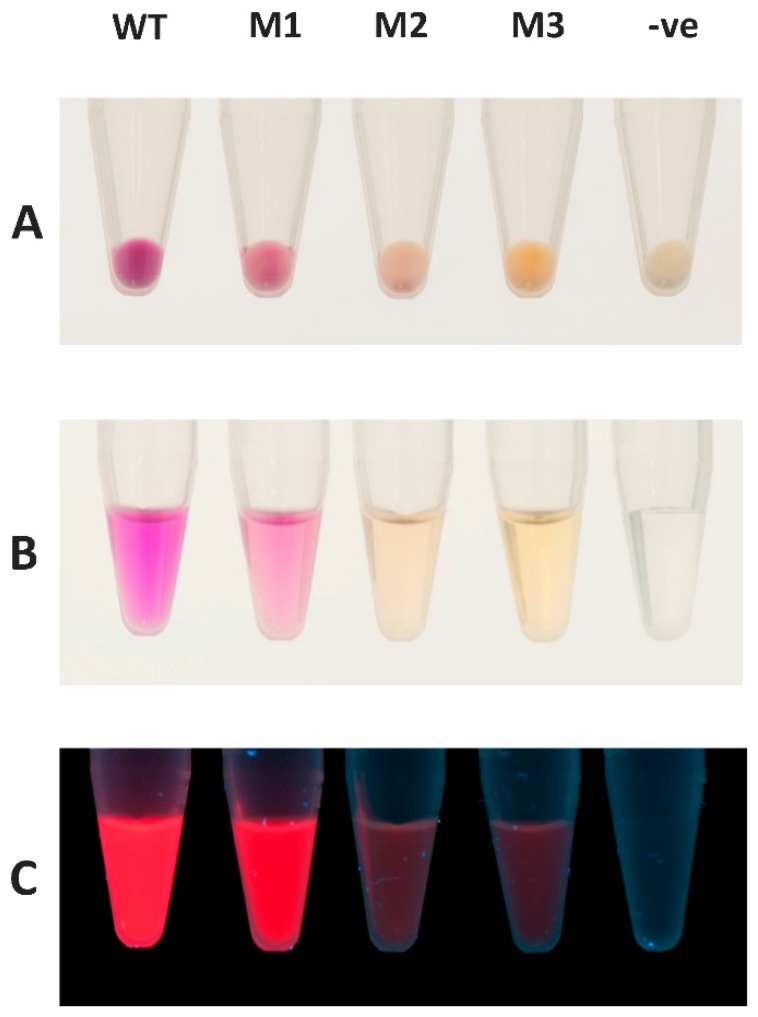
Comparison between mRFP1 wild type (WT) and the three variants (M1, M2, and M3) isolated from the PTO-QuickStep library. (**A**) Cell pellets of *E. coli* C41 (DE3) expressing the red fluorescent proteins. (**B**) Proteins purified using Ni-NTA Spin Columns (Qiagen). (**C**) Purified proteins under UV light. (-ve) denotes negative control, i.e., *E. coli* C41 (DE3) without a plasmid.

**Figure 5:**
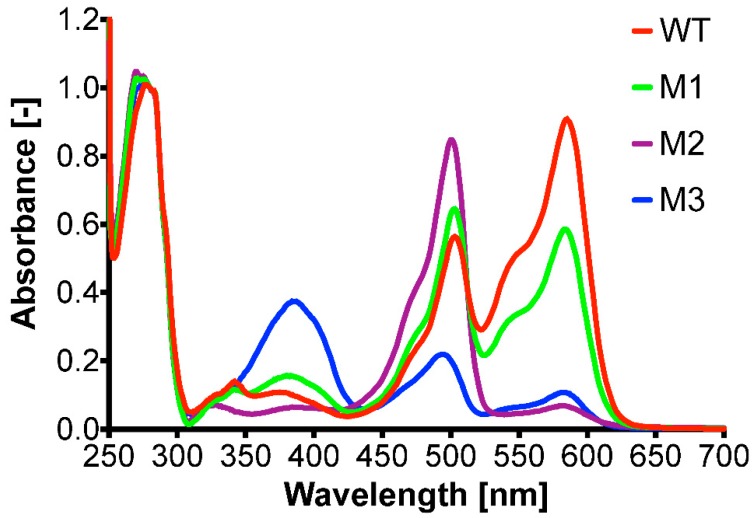
Normalized absorption spectra of mRFP1 wild type (WT; red) and the three variants [M1 (green), M2 (purple), and M3 (blue)].

Three mRFP1 protein variants exhibiting a different color under visible light were isolated from the PTO-QuickStep *rfp* library for further characterization (Figure 4). These variants displayed different absorption spectra (Figure 5). The purified mRFP1 wild type showed an excitation maximum of 585 nm, consistent with previously reported values [16,19,20]. All variants (M1–M3) had blue-shifted excitation maxima. M1, the brightest variant of all, had two absorption peaks of almost equal height at 503 nm and 584 nm.

DNA sequencing revealed nonsynonymous mutations in all three variants (Table 3). In fact, the profile of nucleotide substitutions obtained (A→T, A→G, T→C) was consistent with the expected mutational spectrum of the epPCR condition chosen [21]. Interestingly, two of the four mutated amino acid positions (Q66 and T195) are also the key determinants in creating DsRed variants (e.g., dTomato [16], mCherry [16], mStrawberry [16], mTangerine [16], mOrange [16], mBanana [16], mHoneydew [16], pHTomato [22], and an improved mRFP1 variant reported by Jach et al. [19]). Therefore, PTO-QuickStep enabled fast and efficient creation of good-quality mutant library for directed evolution.

### 2.6. PTO-QuickStep: Potential Limitations and Mitigation

PTO-QuickStep is a significant advancement of the established QuickStep method for seamless cloning of either a specific DNA sequence or a gene library for directed evolution. Therefore, PTO-QuickStep is expected to facilitate day-to-day molecular cloning and expedite the directed evolution workflow. As demonstrated in this article, PTO-QuickStep can easily be coupled with different transformation methods, depending on application and need. For example, electroporation generates a larger library for directed evolution experiment. Potential means to further increase the library size include the use of commercially available ultracompetent cells or treating the PTO-QuickStep product with nick-repairing enzymes prior to transformation (e.g., New England Biolabs’ PreCR Repair Mix).

As is the case with most PCRs or PCR-based methods, the success hinges on the correct primer design. PTO-QuickStep requires only a pair of PTO-modified primers compared to four primers needed for QuickStep. This change should minimize the chances of incorrect primer design. As PTO chemistry is one of the cheapest oligonucleotide modifications available, both primer configurations have the same price (£12 for either a pair of desalted PTO primers used for RFP-cloning experiments or four desalted unmodified oligos needed for the original QuickStep, as given by Eurofins Genomics).

It should be pointed out that both PTO-QuickStep and QuickStep are optimized for point insertion of DNA. They are not the methods of choice for gene replacement. Furthermore, the method has not been tested for simultaneous cloning of multiple fragments, although there is a possibility of incorporating an overlapping PCR into the method to create a megaprimer composed of several fragments.

A comparison between the *rfp* and *cpr* cloning experiments (Table 2 and Figure 3) indicates that the cloning efficiency decreases with increasing length of either the target gene or the recipient plasmid, with the latter appearing to have a more profound effect. This is often observed with methods relying on whole-plasmid amplification, such as the QuikChange protocols for focused mutagenesis. As such, for more challenging cloning experiments (for example, when a target gene is longer than 2 kb), a standard CaCl_2_ chemical transformation protocol might prove insufficient, and the use of a more efficient transformation method (e.g., electroporation or commercially available competent cells) is therefore advised.

## 3. Materials and Methods

### 3.1. Materials

All enzymes, deoxyribonucleotides, and DNA ladders were purchased from New England Biolabs Ltd. (Hitchin, UK).

### 3.2. Primers

All primers used in this study (Table 1) were synthesized by Eurofins Genomics (Ebersberg, Germany). The melting temperatures of oligonucleotides were determined using the NEB Tm calculator (https://tmcalculator.neb.com/#!/main).

### 3.3. QuickStep-Cloning

To clone *rfp* gene from pBbA8k-RFP into pET24a-HLTev-p53, two asymmetric PCRs were carried out in parallel. Asymmetric PCR mixture I (50 µL) contained 1× Q5 reaction buffer, 200 µM of each dNTP, 500 nM *RFP-Fwd* primer, 10 nM *IntB-RFP-Rev* primer, 0.2 ng pBbA8k-RFP, and 1 U Q5 High-Fidelity DNA Polymerase. Asymmetric PCR mixture II (50 µL) contained 1× Q5 reaction buffer, 200 µM of each dNTP, 10 nM *IntA-RFP-Fwd* primer, 500 nM *RFP-Rev* primer, 0.2 ng pBbA8k-RFP, and 1 U Q5 High-Fidelity DNA Polymerase. Both mixtures were thermocycled using the following conditions: (i) 30 s initial denaturation at 98 °C and (ii) 30 cycles of 7 s denaturation at 98 °C, 20 s annealing at 68 °C, and 30 s extension at 72 °C. The two PCR products were purified using QIAquick PCR Purification Kit (Qiagen, Manchester, UK), and their DNA concentrations were determined using VersaWave Microvolume Spectrophotometer (Expedeon, Cambridge, UK). For megaprimer PCR, the mixture (50 µL) contained 1× Q5 reaction buffer, 200 µM of each dNTP, 200 ng of purified asymmetric PCR product I, 200 ng of purified asymmetric PCR product II, 20 ng pET24a-HLTev-p53, and 1 U Q5 High-Fidelity DNA Polymerase. The mixture was thermocycled using the following conditions: (i) 30 s initial denaturation at 98 °C; (ii) 25 cycles of 10 s denaturation at 98 °C, 4 min annealing and extension at 72 °C; and (iii) 2 min final extension at 72 °C. Forty units of DpnI were subsequently added to the PCR mixture and incubated at 37 °C for 15 min to remove the parental plasmids.

### 3.4. Restriction-Free (RF) Cloning

PCR mixture (50 µL) containing 1× Q5 reaction buffer, 200 µM of each dNTP, 500 nM *IntA-RFP-Fwd* primer, 500 nM *IntB-RFP-Rev* primer, 0.2 ng pBbA8k-RFP, and 1 U Q5 High-Fidelity DNA Polymerase was thermocycled using the same program as the asymmetric PCR in QuickStep-Cloning: (i) 30 s initial denaturation at 98 °C and (ii) 30 cycles of 7 s denaturation at 98 °C, 20 s annealing at 68 °C, and 30 s extension at 72 °C. The PCR product was purified using QIAquick PCR Purification Kit (Qiagen), and its DNA concentration was determined using VersaWave Microvolume Spectrophotometer. Megaprimer PCR mixture (50 µL) containing 1× Q5 reaction buffer, 200 nM of each dNTP, 400 ng of purified PCR product, 20 ng pET24a-HLTev-p53, and 1 U Q5 High-Fidelity DNA Polymerase was thermocycled using the following conditions: (i) 30 s initial denaturation at 98 °C; (ii) 25 cycles of 10 s denaturation at 98 °C, 4 min annealing and extension at 72 °C; and (iii) 2 min final extension at 72 °C. Forty units of DpnI were added to the PCR mixture and incubated at 37 °C for 15 min to remove the parental plasmids.

To clone P450 BM-3 reductase gene (*cpr*) from pCWori-BM3 into pETM11-BMP-WT, the same protocol was followed using a dedicated primer set (*IntA-CPR-Fwd* and *IntB-CPR-Rev*). For the first PCR, the following thermocycling conditions were used: (i) 30 s initial denaturation at 98 °C and (ii) 30 cycles of 7 s denaturation at 98 °C, 80 s annealing and extension at 72 °C. The PCR product was purified as described above. Subsequently, megaprimer PCR mixture (50 µL) containing 1× Q5 reaction buffer, 200 nM of each dNTP, 500 ng of purified PCR product, 25 ng pETM11-BMP-WT, and 1 U Q5 High-Fidelity DNA Polymerase was thermocycled using the following conditions: (i) 30 s initial denaturation at 98 °C; (ii) 25 cycles of 10 s denaturation at 98 °C, 20 s annealing at 66 °C, and 4 min extension at 72 °C; and (iii) 2 min final extension at 72 °C. Forty units of DpnI were added to the PCR mixture and incubated at 37 °C for 15 min to remove the parental plasmids.

### 3.5. PTO-QuickStep

PCR mixture (50 µL) containing 1× Q5 reaction buffer, 200 µM of each dNTP, 500 nM *IntA-RFP-Fwd-PTO* primer, 500 nM *IntB-RFP-Rev-PTO* primer, 0.2 ng pBbA8k-RFP, and 1 U Q5 High-Fidelity DNA Polymerase was thermocycled using the same program as the asymmetric PCR in QuickStep-Cloning: (i) 30 s initial denaturation at 98 °C and (ii) 30 cycles of 7 s denaturation at 98 °C, 20 s annealing at 68 °C, and 30 s extension at 72 °C.

After PCR, 6.25 µL of 0.5 M Tris-HCl buffer (pH 9) and 6.25 µL of 100 mM iodine in absolute ethanol were added directly to the PCR product. After brief mixing by pipetting, the mixture was incubated for 5 min at 70 °C and then snap-cooled on ice.

Subsequently, the mixture was purified using QIAquick PCR Purification Kit (Qiagen), and its DNA concentration was determined using VersaWave Microvolume Spectrophotometer. Megaprimer PCR mixture (50 µL) containing 1× Q5 reaction buffer, 200 nM of each dNTP, 100 ng of purified PCR product, 20 ng pET24a-HLTev-p53, and 1 U Q5 High-Fidelity DNA Polymerase was thermocycled using the same program as the QuickStep-Cloning megaprimer PCR: (i) 30 s initial denaturation at 98 °C; (ii) 25 cycles of 10 s denaturation at 98 °C, 4 min annealing and extension at 72 °C; and (iii) 2 min final extension at 72 °C. Forty units of DpnI were added to the PCR mixture and incubated at 37 °C for 15 min to remove the parental plasmids.

To clone P450 BM-3 reductase gene (*cpr*) from pCWori-BM3 into pETM11-BMP-WT, the same protocol was followed using a dedicated primer set (*IntA-CPR-Fwd-PTO* and *IntB-CPR-Rev-PTO*). For the first PCR, the following thermocycling conditions were used: (i) 30 s initial denaturation at 98 °C and (ii) 30 cycles of 7 s denaturation at 98 °C, 80 s annealing and extension at 72 °C. The PCR product was purified as described above. Subsequently, megaprimer PCR mixture (50 µL) containing 1× Q5 reaction buffer, 200 nM of each dNTP, 100 ng of purified PCR product, 25 ng pETM11-BMP-WT, and 1 U Q5 High-Fidelity DNA Polymerase was thermocycled using the following conditions: (i) 30 s initial denaturation at 98 °C; (ii) 25 cycles of 10 s denaturation at 98 °C, 20 s annealing at 66 °C, and 4 min extension at 72 °C; and (iii) 2 min final extension at 72 °C. Forty units of DpnI were added to the PCR mixture and incubated at 37 °C for 15 min to remove the parental plasmids.

### 3.6. DNA Gel Electrophoresis

PCR products were analyzed using 0.7% tris-borate-ethylenediaminetetraacetic acid (TBE) gel. For visualization purposes, ethidium bromide was added to the gel. Quick-Load 1 kb DNA Ladder was used as a DNA ladder.

### 3.7. Chemical Transformation and Clone Analysis

*E. coli* DH5α and C41 (DE3) were transformed with 5 µL of DpnI-digested products of QuickStep-Cloning, RF cloning, or PTO-QuickStep using a standard CaCl_2_ chemical transformation protocol. Concurrently, the bacterial strains were transformed with 1 ng of intact pET24a-HLTev-p53 or pETM11-BMP-WT to estimate the transformation efficiency. Transformed bacteria were plated on TYE agar plates (10 g/L tryptone, 5 g/L yeast extract, 8 g/L sodium chloride, and 15 g/L agar) supplemented with 50 µg/mL kanamycin and 1 mM isopropyl β-D-1-thiogalactopyranoside (IPTG) (only for *rfp* cloning experiment). The plates were incubated overnight at 37 °C and for a further 12 h at 30 °C. The number of mRFP1-expressing colonies was determined by visual inspection using UV transilluminator.

### 3.8. Error-Prone PCR

PCR mixture (50 µL) containing 1× Standard Taq (Mg-free) reaction buffer, 7 mM MgCl_2_, 0.05 mM MnCl_2_, 200 µM of deoxyadenosine triphosphate (dATP), 200 µM of deoxyguanosine triphosphate (dGTP), 1 mM of deoxythymidine triphosphate (dTTP), 1 mM of deoxycytidine triphosphate (dCTP), 400 nM *IntA-RFP-Fwd-PTO* or *IntA-RFP-Fwd* primer (depending on whether PTO-QuickStep or MEGAWHOP was used, respectively), 400 nM *IntB-RFP-Rev-PTO* or *IntB-RFP-Rev* primer, 50 ng pBbA8k-RFP, and 1.25 U Taq DNA Polymerase was thermocycled using the same program as the asymmetric PCR in QuickStep-Cloning: (i) 30 s initial denaturation at 95 °C; (ii) 30 cycles of 20 s denaturation at 95 °C, 30 s annealing at 55 °C, and 45 s extension at 68 °C; and (iii) final extension at 68 °C for 5 min. The PCR product was purified using QIAquick PCR Purification Kit (Qiagen), and its DNA concentration was determined using VersaWave Microvolume Spectrophotometer.

### 3.9. MEGAWHOP

Megaprimer PCR mixture (50 µL) containing 1× Q5 reaction buffer, 200 nM of each dNTP, 500 ng megaprimer, 50 ng pET24a-HLTev-RFP-p53, and 1 U Q5 High-Fidelity DNA Polymerase was thermocycled using the following conditions: (i) 30 s initial denaturation at 98 °C; (ii) 25 cycles of 10 s denaturation at 98 °C, 4 min annealing and extension at 72 °C; and (iii) 2 min final extension at 72 °C. Forty units of DpnI were added to the PCR mixture and incubated at 37 °C for 15 min to remove the parental plasmids.

### 3.10. Transformation of mRFP1 Library

The products of PTO-QuickStep and MEGAWHOP were purified using QIAquick PCR Purification Kit (Qiagen). One microliter of the purified mixture was used to transform *E. coli* C41 (DE3) cells using a standard electroporation protocol. After the transformation, the cells were plated at different dilutions on Luria–Bertani (LB) agar plates (10 g/L tryptone, 5 g/L yeast extract, 10 g/L sodium chloride, and 15 g/L agar) supplemented with 50 µg/mL kanamycin and 1 mM IPTG. The plates were incubated overnight at 37 °C and for a further 12 h at 30 °C. Three colonies exhibiting a different color under visible light compared to that of the mRFP1 wild type were identified by visual inspection and were grown overnight at 37 °C in 5 mL 2× TY medium (16 g/L tryptone, 10 g/L yeast extract, and 5 g/L NaCl) supplemented with 50 µg/mL kanamycin.

### 3.11. mRFP1 Expression and Purification

Overnight cultures (250 µL) of *E. coli* C41 (DE3) corresponding to mRFP1 wild type and the three isolated variants were used to inoculate 50 mL LB medium supplemented with 50 µg/mL kanamycin and grown at 37 °C with shaking. When OD_600_ reached 0.6, the expression was induced with 1 mM IPTG. The cells were grown for a further 16 h. The proteins were purified using Ni-NTA Spin Columns (Qiagen) according to the manufacturer’s instructions. The absorbance measurements were conducted using UV-1600PC UV-VIS spectrophotometer (VWR International, Lutterworth, UK).

## 4. Conclusions

By applying two PTO modifications and strategically placing these PTO bonds within the PCR primers, we streamlined the workflow of QuickStep, enhanced its efficiency, and extended its application to directed evolution. PTO-QuickStep would be of great interest to the synthetic biology community as it facilitates fast, flexible, and efficient construction of recombinant plasmids. This study has also demonstrated that PTO-QuickStep is expected to make a significant contribution to the field of protein engineering.

## Figures and Tables

**Table 1:** Primers used in this study (* represents the location of PTO modification, and DNA sequence in lowercase denotes the region complementary to the recipient plasmid).

Name	Length [bp]	Sequence (5′→3′)
*RFP-Fwd*	19	ATGGCGAGTAGCGAAGACG
*RFP-Rev*	21	TTAAGCACCGGTGGAGTGACG
*IntA-RFP-Fwd*	47	cgaaaacctgtacttccagggtggatccATGGCGAGTAGCGAAGACG
*IntB-RFP-Rev*	47	ctaggatctgactgcggctcctccatTTAAGCACCGGTGGAGTGACG
*IntA-RFP-Fwd-PTO*	47	cgaaaacctgtact*tccagggtggatcc*ATGGCGAGTAGCGAAGACG
*IntB-RFP-Rev-PTO*	47	ctaggatctgact*gcggctcctccat*TTAAGCACCGGTGGAGTGACG
*IntA-CPR-Fwd*	56	ggtaaaagcaaaatcgaaaaaaattccgcttGGCGGTATTCCTTCACCTAGCACTG
*IntB-CPR-Rev*	43	gtcgacggagctcgaattcttaCCCAGCCCACACGTCTTTTGC
*IntA-CPR-Fwd-PTO*	56	ggtaaaagcaaaatc*gaaaaaaattccgctt*GGCGGTATTCCTTCACCTAGCACTG
*IntB-CPR-Rev-PTO*	43	gtcgacggagc*tcgaattctta*CCCAGCCCACACGTCTTTTGC

**Table 2:** Comparison of cloning efficiencies: QS: QuickStep; RF: restriction-free (RF) cloning; PTO-QS: PTO-QuickStep (*Numbers in bracket represent RFP-expressing clones; **intact recipient plasmids were used as reference plasmids; N/A = not applicable; N/D = not determined).

Target Gene/ Recipient Plasmid	Strain/ Transformation Method	QS*	RF*	PTO-QS (25 cycles)*	PTO-QS (30 cycles)*	MEGAWHOP	Transformation Efficiency [cfu/µg]**
*rfp*/ pET24a-HLTev-p53	*E. coli* DH5α/ Chemical	9 (9)	0 (0)	19 (16)	43 (41)	N/A	2.0 × 10^4^
*cpr*/ pETM11-BMP-WT	*E. coli* DH5α/ Chemical	N/D	0	49	N/D	N/A	4.0 × 10^4^
*rfp* library/ pET24a-HLTev-p53	*E. coli* C41 (DE3)/ Electroporation	N/D	N/A	1.0 × 10^4^	N/D	1.5 × 10^4^	2.0 × 10^6^

**Table 3:** Nucleotide and amino acid substitutions found in the three isolated RFP variants (M1, M2, and M3).

RFP Variants	Nucleotide Substitution	Amino Acid Substitution
M1	**A**CC → **T**CC	T195S
M2	**T**TC → **C**TC	F91L
M3	C**A**G → C**T**G	Q66L
	**A**CC → **G**CC	T202A

## Abbreviations

bp	base pair
cfu	colony-forming unit
DNA	deoxyribonucleic acid
dATP	deoxyadenosine triphosphate
dCTP	deoxycytidine triphosphate
dGTP	deoxyguanosine triphosphate
dNTP	deoxynucleoside triphosphate
dTTP	deoxythymidine triphosphate
epPCR	error-prone polymerase chain reaction
IPTG	isopropyl β-D-1-thiogalactopyranoside
kb	kilobase
LB	Luria–Bertani
MEGAWHOP	megaprimer PCR of whole plasmid
PCR	polymerase chain reaction
PTO	phosphorothioate
RF	restriction-free
RFP	red fluorescent protein
ssDNA	single-stranded deoxyribonucleic acid
TBE	tris-borate-ethylenediaminetetraacetic acid
